# Early onset, genital, and axillary involvement are strongly associated with impaired quality of life in hyperhidrosis patients

**DOI:** 10.1097/MD.0000000000047698

**Published:** 2026-02-20

**Authors:** Nasser M. AbuDujain, Yazeed Alrodiman, Leena Allabboudy, Qais A. Almuhaideb, Khalid M. Alghamdi

**Affiliations:** a University Family Medicine Center, King Saud University Medical City, King Saud University, Riyadh, Saudi Arabia; b College of Medicine, Alfaisal University, Riyadh, Saudi Arabia; c Department of Dermatology, King Faisal Specialist Hospital & Research Centre, Riyadh, Saudi Arabia; d Division of Dermatology, McGill University, Montréal, Québec, Canada; e Department of Dermatology, College of Medicine, King Saud University, Riyadh, Saudi Arabia; f Vitiligo Research Chair, College of Medicine, King Saud University, Riyadh, Saudi Arabia.

**Keywords:** association, HDSS, HidroQoL, hyperhidrosis, quality of life, quality of life burden

## Abstract

Hyperhidrosis is a chronic condition marked by excessive sweating that significantly affects patients’ quality of life (QoL). However, this issue is both underrecognized and undertreated, and its physical, emotional, and social burdens are often underestimated, particularly in Middle Eastern populations. A cross-sectional, analytical study was conducted across Saudi Arabia between May 2024 and April 2025. Participants were recruited through dermatology clinics and a Telegram support group. They completed an online self-administered Arabic questionnaire regarding demographics, medical history, the Arabic Hyperhidrosis Disease Severity Scale, and the Hyperhidrosis Quality of Life Index. A total of 276 Arabic-speaking adults with hyperhidrosis participated. The mean age was 27.3 years (SD ± 8.2), and 57.6% of the respondents were male. Most participants (82.9%) had severe hyperhidrosis (Hyperhidrosis Disease Severity Scale grades 3–4). The mean HidroQoL scores were 10.3 out of 14 for daily life and 16.6 out of 22 for psychosocial life, indicating moderate to severe burden. Disease severity showed the strongest association with QoL impairment (*P* = .001). Other factors significantly associated with daily functioning included lower education level, lower income, and sweating in the axillae and genital region, while genital involvement and low education were associated with psychosocial distress. Hyperhidrosis imposed a substantial QoL burden, especially in patients with severe symptoms and lower socioeconomic status. The findings supported the need for early detection and tailored treatment strategies and the use of culturally validated patient-reported outcome measures like the Arabic HidroQoL in clinical practice.

## 1. Introduction

Hyperhidrosis (HH) is a chronic condition marked by excessive sweating that exceeds the body’s need for thermoregulation, Prevalence estimates of HH vary widely worldwide depending on study methods and populations. However, many studies consistently report around 3%, making HH a common but often overlooked condition. HH is classified as primary (idiopathic, usually localized) or secondary (caused by medications or systemic diseases). The localized type is most common, often affecting the armpits, palms, and soles.^[1–3]^

Primary HH often presents in childhood or adolescence, typically before the age of 25. It is characterized by bilateral, symmetrical sweating that commonly involves the axillae, palms, soles, or craniofacial region. Symptoms are usually worse during the day, often exacerbated by stress or heat, and can markedly interfere with daily functioning and social interactions.^[4,5]^ Secondary HH, by contrast, is more commonly generalized and can result from systemic or endocrine disorders such as diabetes, hyperthyroidism, or infections, as well as from pharmacological agents including certain antidepressants and hypoglycemic drugs.^[1]^ Distinguishing between these 2 types is essential because treatment strategies and prognosis differ significantly.

Although not life-threatening, HH significantly impacts one’s quality of life (QoL), affecting physical comfort, emotional well-being, interpersonal relationships, and occupational functioning.^[2]^ Many patients report embarrassment, anxiety, or social withdrawal due to visible perspiration and the perceived stigma associated with the condition. Available treatment options range from topical antiperspirants and botulinum toxin injections to systemic therapies, device-based approaches, and surgical interventions such as endoscopic thoracic sympathectomy, though outcomes and side effects vary.^[4,5]^

Globally, the reported prevalence of HH varies widely, with most studies confirming a strong association between HH and impaired QoL.^[6]^ In India, for example, nearly 18% of dermatology patients reported hyperhidrosis, but only a minority were diagnosed or received treatment, underscoring the condition’s under-recognition.^[7]^ In the Middle East, studies similarly point to a high prevalence but also highlight dissatisfaction with current therapies, as transient effects and adverse reactions often lead to treatment discontinuation and psychological distress.^[8]^

In Saudi Arabia, HH has emerged as a notable public health concern. A study from Al-Ahsa City reported that 18.3% of participants experienced disruptive sweating, which significantly interfered with their daily activities.^[3]^ Local surgical studies, including those on thoracoscopic sympathectomy, demonstrated high satisfaction rates and improved QoL, even when compensatory sweating was present.^[9,10]^ More recently, the validation of the Arabic Hyperhidrosis Quality of Life Index (HidroQoL) has provided a culturally adapted tool for measuring disease burden and advancing patient-centered care in the Arab region.^[11]^

Assessing the QoL impact and identifying factors associated with disease burden are essential for improving patient-centered care and optimizing treatment decisions. In this vein, patient-reported outcome measures (PROMs), such as self-administered questionnaires, have been progressively used in clinical settings to assess the subjective experiences of individuals with chronic conditions.^[12]^ Several PROMs have been developed specifically for hyperhidrosis, including the Hyperhidrosis Quality-of-Life Questionnaire, the Hyperhidrosis Impact Questionnaire, the Hyperhidrosis Questionnaire, and the increasingly validated HidroQoL.^[13]^ However, only a few studies in Saudi Arabia have applied these tools in conjunction with severity measures to evaluate factors associated with burden, highlighting the need for locally relevant research.

Considering the scarcity of local research, this study offers a significant, comprehensive evaluation of how HH affects daily functioning and psychosocial well-being using the Arabic version of the HidroQoL and objective severity measures such as the Hyperhidrosis Disease Severity Scale (HDSS). This work identifies factors (e.g., symptom severity or income) most strongly associated with QoL and psychosocial well-being. It assesses the QoL burden related to HH and highlights variables that are most influential in this Saudi population. Moreover, future studies should build on these findings through longitudinal research and treatment-based evaluations to better understand causal relationships and improve care strategies and outcomes for affected individuals.

Given the subjective nature of disease burden in HH, this study employed validated PROMs, including the Arabic Hyperhidrosis Disease Severity Scale (Ar-HDSS) and the HidroQoL, to assess symptom severity and QoL from the patient’s perspective. While these measures provide valuable insight into personal experiences and symptom burden, they complement rather than replace clinical assessments by dermatologists using standardized criteria. Therefore, this study’s objectives are to assess the subjective severity of HH and the impact of HH (predominantly primary HH) on QoL among native Arabic-speaking adults in Saudi Arabia, using validated questionnaires, and to examine the associations between disease severity, demographic factors, and QoL impairment.

## 2. Methodology

### 2.1. Study design, participants, and setting

A quantitative, analytical, cross-sectional study was conducted across Saudi Arabia from May 2024 to April 2025. An Arabic self-administered questionnaire was shared with participants through a hyperhidrosis support group on the Telegram application and distributed at dermatology clinics at King Saud University Medical City in Riyadh, Saudi Arabia.

Only native Arabic speakers were included to ensure accurate comprehension and interpretation of the survey items, which were administered in Arabic. To further maintain methodological rigor, participants were eligible if they: self-reported a diagnosis of hyperhidrosis; were aged 18 years or older; were fluent Arabic speakers to ensure accurate understanding of the validated Ar-HDSS and Arabic HidroQoL instruments; and were residents of Saudi Arabia during the study period. Individuals were excluded if they were non-Arabic speakers or younger than 18 years. These criteria ensured that the sample reflected adults with chronic hyperhidrosis and that the administration of Arabic PROMs was appropriate and valid.

### 2.2. Sample size estimation

Because no prior Saudi national data using the HidroQoL instrument were available, the sample size was estimated using the standard approach for studies with an unknown population size. Assuming a 95% confidence level, a conservative expected proportion of 50% (used when prevalence is unknown), and a margin of error of 6%, the calculated minimum required sample was approximately 267 participants.

This target was also consistent with feasibility benchmarks from previous hyperhidrosis quality-of-life studies, which typically include 150 to 300 participants and are adequately powered to detect moderate associations in multivariable analyses. Accordingly, we aimed for a sample of about 250 to 270 participants to ensure sufficient precision and allow adjustment for multiple demographic and clinical predictors.

A total of 276 complete responses were ultimately obtained, exceeding the minimum requirement and meeting recommended thresholds for stable parameter estimation in regression models (e.g., 10–15 participants per predictor variable). This sample size was therefore considered robust and appropriate for the analytical objectives of the study.

### 2.3. Questionnaire

Patients enrolled in the study completed an online survey comprising 3 sections: demographic information; past medical history for their HH; and a validated instruments in HH QoL and the severity assessments. The demographic section collected data on age, sex, level of education, occupation, and monthly income. The medical history section involved the past medical history of chronic medical illness, age of diagnosis with HH, affected sites, and therapeutic interventions used. The last section contained the validated Arabic translations of the HDSS and the HidroQol.

The questionnaire was administered via SurveyMonkey and distributed through a Telegram hyperhidrosis support group and dermatology clinics at King Saud University Medical City in Riyadh. Participation was voluntary, anonymous, and open to individuals with a self-reported diagnosis of hyperhidrosis who were native Arabic speakers. Only fully completed responses were included in the analysis. While no login or IP tracking was employed, multiple entries were minimized by screening data for anomalies. Due to the open nature of recruitment, specific participation and view rates could not be calculated.

#### 2.3.1. Hyperhidrosis Disease Severity Scale

The HDSS was created by Kowalski et al in 2004 as a self-assessment instrument. This tool evaluates the degree of hyperhidrosis a patient experiences using this question: “How would you rate the severity of your hyperhidrosis?.”^[14,15]^ It demonstrates good validity measures (kappa coefficient of 0.732, and *R* = 0.425 with Arabic HidroQoL).

#### 2.3.2. Hyperhidrosis QoL index

Developed by P. Kamudoni in 2015, the HidroQoL is a self-reported questionnaire that measures the impact of hyperhidrosis on QoL. It comprises 18 questions divided into 2 categories: daily activities and psychosocial aspects. Each question is scored using a three-point scale: “Not at all” (0 points), “A little” (1 point), and “Very much” (2 points). When all item scores are summed, the overall total can range from 0 to 36. Total scores are used to assess the severity of the disease’s effect. Scores 0 to 1 mean there is no impact, 2 to 11 indicate a mild effect, 12 to 22 reflect a moderate effect, 23 to 32 show a substantial effect, and 33 to 36 reveal a severe effect.^[12,16,17]^

The HidroQoL instrument has undergone validation and reliable testing, confirming its effectiveness in quantifying disease burden on both daily life and psychosocial functioning. Each domain (activities of daily living and psychosocial aspects) also permits separate scoring, helping clinicians understand and track the wide-ranging effects of hyperhidrosis. This tool is available in culturally adapted versions, such as an Arabic edition, and has proved to maintain strong reliability and validity in various populations It demonstrates good validity measures (kappa coefficient of 0.732, and *R* = 0.425 with Arabic HidroQoL).^[11]^

### 2.4. Ethical considerations

Ethical clearance for this investigation was formally granted by the Institutional Review Board of the College of Medicine, King Saud University, in February 2024. Prior to participation, individuals affirmed their willingness to take part through electronic consent, having been thoroughly apprized of the study’s objectives, anticipated duration, the principal investigator’s contact details, and their unequivocal right to withdraw at any stage without consequence. To safeguard confidentiality, no identifying information was recorded, thereby preserving participant anonymity. Moreover, participation was undertaken purely voluntarily, as no financial or material incentives were offered.

### 2.5. Data analysis

Data analysis was conducted using Statistical Package for the Social Sciences version 28 (IBM Corp., 2021; Armonk). Descriptive statistics, including frequencies, means, and standard deviations, were used to summarize the demographic characteristics and responses of the participants. The assumption of normality was considered based on sample size, shape of distribution, and distribution characteristics. To examine the relationship between hyperhidrosis severity and QoL, the researchers performed a one-way analysis of variance, testing for differences in QoL scores across varying levels of severity. To assess the impact of hyperhidrosis on QoL, multiple linear regression was applied to identify factors significantly associated with both daily life activities and psychosocial well-being. The regression models included various demographic and clinical variables, such as age, gender, education level, income, comorbidities, affected body areas, and severity of hyperhidrosis. Regression coefficients were reported as both unstandardized and standardized values, along with their respective *t* values and *P*-values, to describe the strength and direction of the associations between these factors and QoL outcomes.

## 3. Results

### 3.1. Demographic and clinical characteristics

Table 1 presents the biodemographic data of 276 individuals diagnosed with hyperhidrosis across Saudi Arabia. The mean age was 27.3 ± 8.2 years, with the largest proportion aged 20 to 24 years (30.1%), followed by 25 to 29 years (26.4%). A majority were male (57.6%) and Saudi nationals (85.9%). Most participants held a university degree (71.0%), and 6.9% had postgraduate qualifications. Regarding employment status, 43.8% were employed outside healthcare, 29.0% were unemployed, 22.5% were students, and 4.7% worked in healthcare. Over half (55.8%) reported a monthly income of <5000 Saudi Riyal.

**Table 1:** Biodemographic characteristics of the study hyperhidrosis cases, Saudi Arabia (N = 276).

Biodemographics	No	%
Age in years		
<20	37	13.4%
20–24	83	30.1%
25–29	73	26.4%
30–39	56	20.3%
40+	27	9.8%
Mean ± SD	27.3 ± 8.2	
Gender		
Male	159	57.6%
Female	117	42.4%
Nationality		
Saudi	237	85.9%
Non-Saudi	39	14.1%
Educational level		
Secondary/below	61	22.1%
University graduate	196	71.0%
Postgraduate	19	6.9%
Work field		
Not working	80	29.0%
Student	62	22.5%
Non-health care field	121	43.8%
Health care field	13	4.7%
Monthly income		
<5000 SR	154	55.8%
5000–15,000 SR	81	29.3%
15,000–30,000 SR	36	13.0%
> 30,000 SR	5	1.8%
Comorbidities		
None	241	87.3%
DM	4	1.4%
HTN	4	1.4%
Hypercholesterolemia	6	2.2%
Hypothyroidism	5	1.8%
Others	16	5.8%
Age at onset of hyperhidrosis		
Since birth	78	28.3%
1–9 years old	61	22.1%
10–15 years old	80	29.0%
16–20 years old	36	13.0%
After 20 years old	21	7.6%
Mean ± SD	9.4 ± 8.7	

Values are presented as frequencies and percentages unless otherwise indicated. Continuous variables are reported as mean ± standard deviation (SD).

DM = diabetes mellitus; HTN = hypertension; SR = Saudi Riyal.

Most participants (87.3%) had no reported comorbidities, but a small percentage of them reported conditions including diabetes mellitus, hypertension, hypercholesterolemia, and hypothyroidism. The average age of onset of hyperhidrosis was 9.4 ± 8.7 years. The most common age of symptom onset was 10 to 15 years (29.0%), closely followed by congenital onset (28.3%), and then onset at ages 1 to 9 (22.1%). Only 7.6% of the participants reported onset after age 20.

### 3.2. Affected areas and treatment modalities

**Figure 1.**
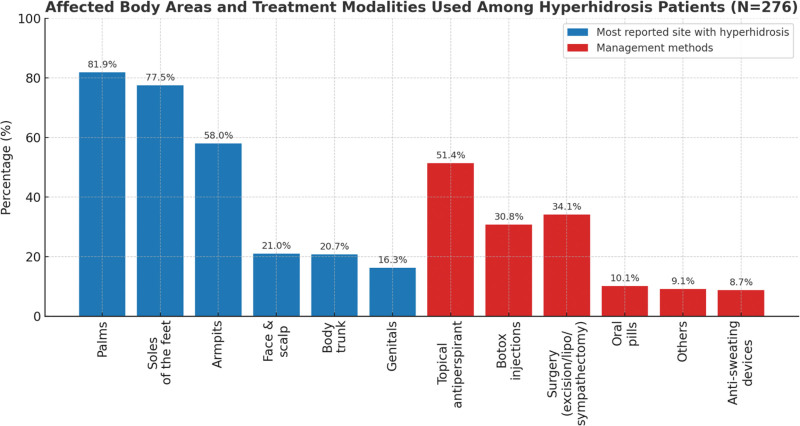
Affected body areas and treatment modalities used among hyperhidrosis patients. HDSS severity levels are displayed as percentages of participants reporting each grade. Higher grades reflect greater interference with daily activities. HDSS = Hyperhidrosis Disease Severity Scale.

Figure 1 illustrates affected body areas and treatment methods. The most commonly involved areas were the palms (81.9%), soles of the feet (77.5%), and armpits (58.0%). Less frequently affected regions included the face/scalp (21.0%), trunk (20.7%), and genital area (16.3%). Regarding treatment, the most widely used method was topical antiperspirants (51.4%); followed by surgical interventions such as skin excision, liposuction, or sympathectomy (34.1%); and Botox injections (30.8%). Less commonly used methods included oral antiperspirants (10.1%), anti-sweating devices (8.7%), and other treatments (9.1%).

### 3.3. Disease severity (HDSS)

**Figure 2.**
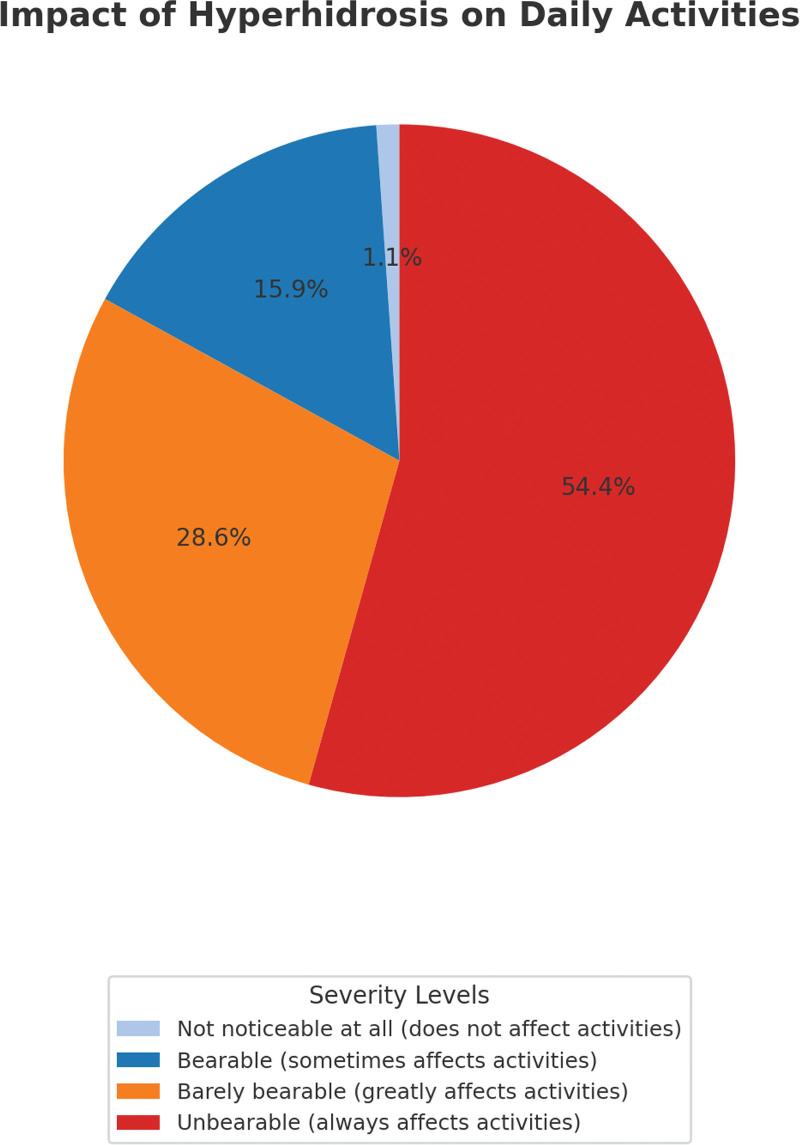
Self-rated severity of hyperhidrosis and its impact on daily activities among participants. HDSS severity levels are displayed as percentages of participants reporting each grade. Higher grades reflect greater interference with daily activities. HDSS = Hyperhidrosis Disease Severity Scale.

Figure 2 shows the distribution of hyperhidrosis severity based on the HDSS. Most participants (54.3%) rated their condition as Grade 4 severity (“unbearable and always affects daily activities”) Another 28.6% reported Grade 3 (“barely bearable and greatly affects daily activities”). Collectively, 82.9% of the sample reported severe disease (HDSS Grades 3–4). Mild to moderate severity (Grade 2) was reported by 15.9%, while only 1.1% indicated minimal impact (Grade 1).

### 3.4. QoL impact

Table 2 summarizes the impact of hyperhidrosis on daily life activities and psychosocial well-being. The mean daily life score was 10.3 out of 14, indicating moderate to severe burden. Over 50% of the participants reported substantial limitations in clothing choices, physical activity, hobbies, and work. Notably, 86.6% expressed concern about managing hyperhidrosis during daily activities, and nearly half reported effects on holidays and physical appearance.

**Table 2:** Quality of life among cases with hyperhidrosis, Saudi Arabia (N = 276).

Items	Not at all	A little	Very much
Daily life activities			
My clothing choices are affected	48 (17.4%)	62 (22.5%)	166 (60.1%)
My physical activity is affected	54 (19.6%)	78 (28.3%)	144 (52.2%)
My hobbies are affected	44 (15.9%)	68 (24.6%)	164 (59.4%)
My work is affected	17 (6.2%)	75 (27.2%)	184 (66.7%)
I worry about dealing with hyperhidrosis when doing extra activities	7 (2.5%)	30 (10.9%)	239 (86.6%)
My holidays are affected (in terms of planning, activities, etc)	59 (21.4%)	82 (29.7%)	135 (48.9%)
My appearance is affected	47 (17.0%)	87 (31.5%)	142 (51.4%)
Min–max (mean ± SD)	0–14 (10.3 ± 3.6)		
Psychosocial life			
I feel stressed	10 (3.6%)	57 (20.7%)	209 (75.7%)
I feel embarrassed	5 (1.8%)	37 (13.4%)	234 (84.8%)
I feel frustrated	27 (9.8%)	63 (22.8%)	186 (67.4%)
I am bothered by physically expressing emotions (like hugging)	46 (16.7%)	55 (19.9%)	175 (63.4%)
Hyperhidrosis is constantly on my mind	12 (4.3%)	48 (17.4%)	216 (78.3%)
I worry about my future health	54 (19.6%)	74 (26.8%)	148 (53.6%)
I worry about how others will react	11 (4.0%)	49 (17.8%)	216 (78.3%)
I worry about leaving sweat stains on things	13 (4.7%)	36 (13.0%)	227 (82.2%)
I avoid meeting new people	60 (21.7%)	80 (29.0%)	136 (49.3%)
I avoid public speaking (like giving presentations)	47 (17.0%)	71 (25.7%)	158 (57.2%)
My sexual life is affected	141 (51.1%)	62 (22.5%)	73 (26.4%)
Min–max (mean ± SD)	1–22 (16.6 ± 4.9)		

Items are reported as frequencies and percentages across 3 response options (not at all, a little, very much). Domain summary scores are expressed as minimum–maximum values with mean ± SD. Daily life domain ranges from 0 to 14; psychosocial domain ranges from 1 to 22. Higher scores indicate greater impairment.

SD = standard deviation.

The psychosocial impact was similarly significant, with a mean score of 16.6 out of 22. Over 75% of respondents experienced high levels of stress, embarrassment, and frustration. Nearly 80% worried about sweat stains or that others would notice their sweat. Many reported avoiding social interactions such as meeting new people or public speaking, emphasizing the condition’s emotional toll.

### 3.5. Association between severity and QoL

As shown in Table 3, an increasing severity of hyperhidrosis was significantly associated with a negative impact on both daily life activities and psychosocial well-being, as assessed by mean QoL scores. Participants with minimal symptoms (Grade 1) had the lowest scores (daily life: 0.67 ± 0.58; psychosocial: 2.33 ± 1.15). In contrast, those with Grade 4 severity had the highest burden (daily life: 11.41 ± 2.94; psychosocial: 17.74 ± 3.97). Intermediate grades showed proportional increases, confirming a graded relationship. These differences were statistically significant (*P* < .001).

**Table 3:** Association between hyperhidrosis severity and quality of life scores in daily life activities and psychosocial well-being.

Severity of hyperhidrosis	Daily life activities	Psychosocial life
	Mean ± SD	Mean ± SD
My sweating is never noticeable and never interferes with my daily activities	0.67 ± 0.58	2.33 ± 1.15
My sweating is tolerable, but sometimes interferes with my daily activities	7.16 ± 3.81	13.59 ± 6.08
My sweating is barely tolerable and frequently interferes with my daily activities	10.15 ± 3.15	16.73 ± 4.40
My sweating is intolerable and always interferes with my daily activities	11.41 ± 2.94	17.74 ± 3.97
*P*-value	.001*	.001*

Values reported as mean ± SD of daily life and psychosocial scores across HDSS severity grades. Statistical comparison performed using one-way ANOVA.

ANOVA = analysis of variance, HDSS = Hyperhidrosis Disease Severity Scale; SD = standard deviation.

* *P* < .05 indicates statistical significance.

### 3.6. Factors associated with daily life impact

Multiple linear regression (Table 4) identified several factors significantly associated with daily life burden. Severity of HH was the strongest associated factor (β = 0.46, *P* = .001). Additional factors included lower educational level (B = ‐1.07, *P* = .004), lower income (B = ‐0.71, *P* = .024), and the presence of hypertension (B = ‐3.39, *P* = .041). Affected anatomical sites (specifically the armpits [B = 1.17, *P* = .004] and genital area [B = 1.25, *P* = .032]) were also associated with a higher burden. Notably, palmar involvement was negatively associated (B = ‐1.56, *P* = .048), possibly reflecting adaptive coping mechanisms. Additionally, older age was associated with greater impact (*P* = .022). The regression model was statistically significant (*P* = .027), indicating that the independent variables included in the model collectively explain a meaningful portion of the variance in the outcome variable. The adjusted *R*^2^ value of 0.39 suggests that approximately 39% of the variability in the dependent variable can be explained by the model, which reflects a moderate explanatory power.

**Table 4:** Multiple linear regression model for factors associated with daily life activities among patients with hyperhidrosis.

Factors	Unstandardized coefficients		Standardized coefficients	*t*	*P*-value
	B	SE	Beta		
Age in years	0.06	0.03	0.15	2.31	.022*
Female gender	‐0.09	0.41	‐0.01	‐0.22	.823
Educational level	‐1.07	0.37	‐0.15	‐2.92	.004*
The healthcare work field	‐0.30	0.24	‐0.08	‐1.25	.214
Monthly income	‐0.71	0.31	‐0.15	‐2.27	.024*
DM	0.56	1.65	0.02	0.34	.737
HTN	‐3.39	1.65	‐0.11	‐2.06	.041*
Hypercholesterolemia	‐0.68	1.29	‐0.03	‐0.53	.599
Hypothyroidism	0.25	1.41	0.01	0.17	.861
Armpit affected area	1.17	0.40	0.16	2.91	.004*
Palms affected area	-1.56	0.63	-0.17	-1.89	.048*
Soles of the feet affected area	0.93	0.77	0.11	1.21	.226
Trunk of the body affected area	-0.46	0.53	-0.05	-0.88	.382
The face and scalp affected area	0.18	0.52	0.02	0.35	.729
Genital area affected area	1.25	0.58	0.13	2.16	.032*
Severity of Hyperhidrosis	2.13	0.23	0.46	9.15	.001*

Unstandardized coefficients (B), standard errors (SE), standardized coefficients (Beta), *t* values, and *P*-values are reported for each predictor.

DM = diabetes mellitus, HTN = hypertension, SE = standard error.

* Model significance set at *P* < .05.

### 3.7. Factors associated with psychosocial impact

Table 5 presents the results of multiple linear regression analysis identifying factors significantly associated with psychosocial impact among patients with hyperhidrosis. Hyperhidrosis severity remained the most significant factor in psychosocial life (B = 2.29, β = 0.37, *P* = .001). Beyond this, lower educational attainment (B = ‐1.36, β = ‐0.14, *P* = .016) and genital area involvement (B = 1.55, β = 0.12, *P* = .049) were associated with worse psychosocial outcomes. Finally, monthly income showed a borderline association with these outcomes (*P* = .060). The regression model was statistically significant (*P* = .039), indicating that the independent variables included in the model collectively explain a meaningful portion of the variance in the outcome variable. The adjusted *R*^2^ value of 0.37 suggests that approximately 37% of the variability in the dependent variable can be explained by the model, which reflects a moderate explanatory power.

**Table 5:** Multiple linear regression model for factors associated with psychosocial life among patients with hyperhidrosis.

Factors	Unstandardized coefficients		Standardized coefficients	*t*	*P*-value
	B	SE	Beta		
Age in years	.05	.04	.08	1.15	.251
Female gender	.38	.62	.04	.61	.544
Educational level	‐1.36	.56	‐.14	‐2.43	.016*
The healthcare work field	.26	.37	.05	.71	.481
Monthly income	‐.89	.47	‐.14	‐1.89	.060
DM	.95	2.52	.02	.38	.705
HTN	‐3.69	2.51	‐.09	‐1.47	.143
Hypercholesterolemia	.92	1.97	.03	.47	.640
Hypothyroidism	‐1.80	2.14	‐.05	‐.84	.401
Armpit affected area	.85	.61	.09	1.39	.165
Palms affected area	‐1.59	1.25	‐.12	‐1.27	.206
Soles of the feet affected area	.80	1.17	.07	.68	.496
Trunk of the body affected area	‐1.07	.81	‐.09	‐1.33	.186
The face and scalp affected area	‐.47	.79	‐.04	‐.60	.550
Genital area affected area	1.55	.78	.12	1.75	.049*
Severity of hyperhidrosis	2.29	.35	.37	6.47	.001*

Regression coefficients (B), SE, beta, *t* values, and *P* values represent associations between patient characteristics and psychosocial burden.

DM = diabetes mellitus, HTN = hypertension, SE = standard error.

* *P* < .05 indicates statistical significance.

## 4. Discussion

A striking 82.9% of patients in our cohort were classified as having severe hyperhidrosis (HDSS grades 3–4), underscoring the significant disease burden in this population. This study highlights the significant impact of hyperhidrosis on both daily functioning and psychosocial well-being. Using the HidroQoL, we observed moderate to severe impairment in both domains, with symptom severity showing the strongest and most consistent association with QoL.

These findings aligned with earlier qualitative and quantitative studies highlighting the broad, disruptive impact of hyperhidrosis on individuals’ lives.^[4,5,12]^ Similar patterns have been described in the literature on primary focal hyperhidrosis, where patients have reported effects on self-esteem, interpersonal relationships, professional performance, and emotional health.^[18]^

Regression analyses confirmed disease severity as the factor most strongly associated with both daily and psychosocial burden. Participants with Grade 4 HDSS scores reported the highest levels of distress, echoing the findings of Hamm et al, who showed that QoL impairment in hyperhidrosis can be as profound as in chronic dermatological conditions such as psoriasis or eczema.^[19]^ This result was also consistent with previous research that has demonstrated a high correlation between hyperhidrosis severity and QoL deterioration.^[4]^ Furthermore, these results validated using HDSS as a quick, reliable proxy for identifying patients with high disease burden. This information mirrored the work of Naumann et al, who observed escalating functional, emotional, and social impairments with increasing sweat severity.^[20]^ Patients in the present study with Grades 3 and 4 HDSS scores had the highest HidroQoL values, reinforcing the clinical utility of HDSS as a triage tool.

The anatomical site of hyperhidrosis significantly influenced its impact. Axillary and genital involvement were particularly associated with reduced daily functioning and increased psychosocial distress. These findings reflected the high social sensitivity of these regions, where visible sweat or odor can produce stigmatization and social withdrawal.^[5]^ These results were further supported by earlier reports highlighting the embarrassment and functional limitation caused by sweating in socially sensitive or physically uncomfortable areas.^[5]^ Notably, in this study, palm involvement (often cited as disabling in occupational settings) was negatively associated with daily life burden in the regression model. Several mechanisms may explain this finding. Firstly, palmar hyperhidrosis often presents earlier in life than other subtypes, which may allow patients more time to develop adaptive coping behaviors such as modifying work and social interactions. Secondly, therapeutic options for palmar involvement (iontophoresis, botulinum toxin injections, and in selected cases sympathectomy) are relatively well established and more widely accessible than for craniofacial disease. This wider availability of treatment may help reduce the long-term burden. Thirdly, the psychosocial distress with hyperhidrosis may be more pronounced in axillary or craniofacial forms, where sweating is highly visible to others, compared with palmar sweating, which (though functionally disabling) can often be concealed. Taken together, these factors may partly explain the lower reported burden among patients with palmar disease. Future research should confirm these observations and investigate whether early treatment or cultural context modifies the relationship between subtype and quality-of-life outcomes.

Socioeconomic status, reflected by education and income, also influenced QoL outcomes. Patients with lower education and income levels had significantly higher impairment scores, suggesting that socioeconomic status influences both symptom perception and coping capacity, along with limited access to treatment. Similarly, this pattern has been observed in broader dermatological research on vulnerable populations facing compounded disease burden due to structural barriers.^[5,17]^

Additionally, participants with more severe symptoms reported higher levels of emotional distress, including embarrassment, frustration, and avoidance of social situations. The mean psychosocial score of 16.6 out of 22 indicated a substantial emotional toll. These findings aligned with previous research linking hyperhidrosis to anxiety, diminished self-esteem, and even suicidal ideation in extreme cases.^[18,20]^ Although psychological outcomes were not the primary focus of this study, the strong association between disease severity and emotional distress highlighted the need for integrating mental health screening and support in hyperhidrosis care, especially for those with severe or socially isolating symptoms. The findings in this study emphasized the importance of using validated PROMs like the HidroQoL and HDSS in routine practice. These instruments are efficient to administer and sensitive to clinical change, and they facilitate shared decision-making between patients and providers.^[12,17]^ Given the strong association found between severity and QoL impairment, clinicians should consider stratifying patients based on HDSS scores and tailoring interventions accordingly. Those with high severity and added psychosocial or socioeconomic risk factors might benefit from a multidisciplinary approach, including pharmacological treatment, psychological support, and peer-based resources.

In many international guidelines, HDSS stratification is explicitly used to guide the management of HH. For example, the American Academy of Family Physicians recommends using the HDSS to assess disease severity and predict treatment response.^[21]^ According to the American Academy of Family Physicians, initial treatment should begin with 20% aluminum chloride, botulinum toxin injections, or iontophoresis. Oral anticholinergics and more invasive surgical options are reserved for patients with severe symptoms who do not respond to first-line therapies.^[21]^ Similarly, the Canadian HH Advisory Committee recommends topical aluminum chloride for mild cases (HDSS = 2) of axillary, palmar, and plantar HH, while more intensive treatments such as botulinum toxin A or iontophoresis are suggested for moderate to severe cases (HDSS = 3 or 4).^[22]^ Incorporating HDSS-based stratification into clinical practice in our setting could help standardize care pathways (so that patients with higher severity are triaged for referral to specialized dermatology services and more aggressive therapies, while those with milder disease might be managed effectively in primary care with topical or simpler modalities).

Two major strengths of this study were the large, diverse patient sample and the application of both descriptive and inferential statistical methods to identify factors associated with burden. However, several limitations should be acknowledged. The cross-sectional design restricts causal inference, and all data were self-reported, which raises the possibility of recall or reporting bias. Additionally, recruitment through a Telegram support group and dermatology clinics may have introduced selection bias, as individuals active in online support groups or receiving specialized care may differ systematically from the broader population with HH. Furthermore, the open, anonymous online survey format did not employ IP tracking or login restrictions, potentially allowing multiple entries despite our efforts to screen for anomalies. The sample was also regionally limited to Saudi Arabia, and the exclusion of non-native Arabic speakers may further limit the generalizability of the findings.

Furthermore, the severity of hyperhidrosis was assessed solely using the Ar-HDSS, a patient-reported instrument. While this tool is validated and widely used, it does not substitute for clinical evaluation by dermatologists using standardized diagnostic criteria. Reliance on self-reported severity alongside quality-of-life measures may introduce methodological bias, as both variables were measured using subjective instruments. Moreover, although the study targeted individuals with primary hyperhidrosis, 12.7% of participants reported comorbidities, suggesting that some cases of secondary hyperhidrosis may have been inadvertently included, as no clinical verification or exclusion was performed. Another limitation of this study is the potential variability in diagnostic accuracy, as not all cases were confirmed by board-certified dermatologists. Diagnoses were based on self-reporting, which may introduce a risk of misclassification.

Together, these factors should be taken into account when interpreting the results. Nevertheless, these findings suggest that clinicians should routinely assess both disease severity and patient context, including socioeconomic and anatomical factors, to optimize management strategies.

## 5. Conclusion

This study reinforced that HH imposes a high QoL burden, particularly among individuals with severe symptoms, lower socioeconomic status, and sweating in socially sensitive areas. The severity of HH, education level, income, and anatomical site of sweating were associated with daily and psychosocial impairment. Incorporating disease-specific QoL tools such as the HidroQoL into clinical evaluation could improve assessment, guide personalized care, and enhance patient outcomes. Some international research has been conducted on this subject, but there has been limited data from Saudi Arabia using culturally validated tools. Therefore, this study addressed that gap and highlighted the value of using instruments like the Arabic HidroQoL to guide personalized care. Future studies should explore longitudinal outcomes and intervention effects across diverse populations.

## Acknowledgments

The authors extend their appreciation to the Deanship of Scientific Research, King Saud University, for funding through the Ongoing Research Funding program: Research Chairs (ORF-RC-2025-3900), King Saud University, Riyadh, Saudi Arabia.

## Author contributions

**Conceptualization:** Nasser M. AbuDujain, Yazeed Alrodiman, Qais A. Almuhaideb, Khalid M. Alghamdi.

**Data curation:** Nasser M. AbuDujain, Yazeed Alrodiman, Leena Allabboudy, Qais A. Almuhaideb.

**Formal analysis:** Nasser M. AbuDujain.

**Funding acquisition:** Khalid M. Alghamdi.

**Investigation:** Nasser M. AbuDujain, Yazeed Alrodiman, Qais A. Almuhaideb.

**Methodology:** Nasser M. AbuDujain, Qais A. Almuhaideb.

**Project administration:** Khalid M. Alghamdi.

**Resources:** Leena Allabboudy.

**Software:** Yazeed Alrodiman.

**Supervision:** Khalid M. Alghamdi.

**Visualization:** Nasser M. AbuDujain.

**Writing – review & editing:** Nasser M. AbuDujain, Qais A. Almuhaideb, Khalid M. Alghamdi.

**Writing – original draft:** Yazeed Alrodiman, Leena Allabboudy.
